# CD19 CAR-Targeted T Cells Induce Long-Term Remission and B Cell Aplasia in an Immunocompetent Mouse Model of B Cell Acute Lymphoblastic Leukemia

**DOI:** 10.1371/journal.pone.0061338

**Published:** 2013-04-09

**Authors:** Marco L. Davila, Christopher C. Kloss, Gertrude Gunset, Michel Sadelain

**Affiliations:** 1 Leukemia Service, Department of Medicine, Memorial Sloan-Kettering Cancer Center, New York, New York, United States of America; 2 Center for Cell Engineering, Memorial Sloan-Kettering Cancer Center, New York, New York, United States of America; 3 Biochemistry, Cell, and Molecular Biology Program, Weill Cornell Graduate School of Medical Sciences, Cornell University, New York, New York, United States of America; 4 Molecular Pharmacology and Chemistry Program, Memorial Sloan-Kettering Cancer Center, New York, New York, United States of America; Saint Louis University School of Medicine, United States of America

## Abstract

Although many adults with B cell acute lymphoblastic leukemia (B-ALL) are induced into remission, most will relapse, underscoring the dire need for novel therapies for this disease. We developed murine CD19-specific chimeric antigen receptors (CARs) and an immunocompetent mouse model of B-ALL that recapitulates the disease at genetic, cellular, and pathologic levels. Mouse T cells transduced with an all-murine CD3ζ/CD28-based CAR that is equivalent to the one being used in our clinical trials, eradicate B-ALL in mice and mediate long-term B cell aplasias. In this model, we find that increasing conditioning chemotherapy increases tumor eradication, B cell aplasia, and CAR-modified T cell persistence. Quantification of recipient B lineage cells allowed us to estimate an in vivo effector to endogenous target ratio for B cell aplasia maintenance. In mice exhibiting a dramatic B cell reduction we identified a small population of progenitor B cells in the bone marrow that may serve as a reservoir for long-term CAR-modified T cell stimulation. Lastly, we determine that infusion of CD8+ CAR-modified T cells alone is sufficient to maintain long-term B cell eradication. The mouse model we report here should prove valuable for investigating CAR-based and other therapies for adult B-ALL.

## Introduction

Precursor B cell acute lymphoblastic leukemia (B-ALL) in adults remains a challenging disease to treat [1]. While complete remission rates are high, overall survival remains low, which indicates that residual disease after standard cytotoxic chemotherapy is an important therapeutic target [2]. A promising direction for novel cancer treatment strategies includes immunotherapies that aim to stimulate tumor-specific immune responses. The proof-in-principle for the therapeutic benefit of targeting leukemia by the immune system comes from the Graft vs. Leukemia (GVL) effect seen in allogeneic stem cell transplants in patients with chronic myelogenous leukemia [3]. However, while there is a GVL effect in B-ALL patients undergoing allogeneic bone marrow transplantation, it is less than that seen in CML patients [4]. Our rationale to engineer a cell therapy targeting B-ALL was in part to generate T cells with enhanced anti-leukemic activity.

We have opened a Phase I clinical trial (NCT01044069) to evaluate the safety of autologous, CD19-targeted T cells as a supplement to cytotoxic chemotherapy for adults with B-ALL [5]. We previously demonstrated that these T cells are efficient at eradicating B cell tumors *in vitro* and *in vivo* using immunodeficient mouse models [6], [7]. Furthermore, we and others [5], [8]–[13] have shown elements of therapeutic benefit by targeting CD19 in patients with indolent B cell malignancies (reviewed in [14]). However, there is a significant need for relevant and physiologic pre-clinical models to serve as a platform for the analysis and optimization of cell-engineered therapies. We therefore developed a syngeneic model of B-ALL in immunocompetent mice by isolating a B-cell leukemia from an Eμ-myc transgenic mouse prone to B cell malignancies. This model resembles B-ALL based on molecular, cellular, and pathologic analyses. In both our trial and pre-clinical models we genetically-engineer T cells with a chimeric antigen receptor (CAR) created by fusing the heavy and light chains of an anti-CD19 antibody to the CD28 and CD3ζ signaling domains of a T cell receptor [6], [7]. T cells are retrovirally-transduced with the CD19-targeted CAR to target the T cells to the pan-specific B cell antigen, CD19. We demonstrate that survival is vastly improved in mice with B-ALL that have been treated with a CD19-targeted cell therapy as a supplement to cytotoxic chemotherapy in comparison to mice treated with cytotoxic chemotherapy alone. We further use this immunocompetent model to evaluate the effect of T cell dose, conditioning chemotherapy, and CD8+ T cell subset on the function of CD19 CAR-targeted T cells.

## Materials and Methods

### Ethics Statement

Animal studies were carried out in accordance with the recommendations in the Guide for the Care and Use of Laboratory Animals of the National Institutes of Health and according to the Memorial Sloan-Kettering Cancer Center Institutional Animal Care and Use Committee. All studies were approved by the Memorial Sloan-Kettering Cancer Center Institutional Animal Care and Use Committee under protocols 08-08-020 and 11-03-009.

### Mice

C57BL/6, Thy1.1, and Eμ-myc transgenic mice were obtained from the Jackson Laboratory (Bar Harbor, ME). OTI-Rag2^−/−^ mice were obtained from Taconic (Hudson, NY).

### Cell line

The Eμ-ALL01 cell line was derived from an Eμ-myc transgenic mouse, which was sacrificed due to a progressive lymphoid malignancy (Supplemental Figure S1a). Culturing of the cell line was performed as described [15]. Briefly, an enlarged axillary lymph node was isolated, processed into a suspension of single cells (Supplemental Figure S1b), and cultured in complete RPMI media supplemented with 10% fetal bovine serum (FBS). The cells were passaged twice a week in 6-well plates until they became transformed, as evidenced by their ability to be subcloned as single cells in 96-well plates. The transformed and subcloned Eμ-ALL01 cell line was frozen and archived at passage number 25.

### RT-PCR

Eμ-ALL01 cells were collected into Trizol (Invitrogen, Carlsbad, CA) and RNA was purified according to the manufacturers' instructions. cDNA synthesis and RT-PCR was performed with a One-Step RT-PCR System with Platinum Taq (Invitrogen). Primers for *β-actin*, *Rag1*, *Rag2*, *VpreB*, *Tdt*, *Igβ*, and *Pax5* have been described [16], [17]. The primers for *Cd19* are CD19-For (GGCAATGTTGTGCTGCCATGCCTCC) and CD19-Rev (ATCTCCTGGCGGGGTCAGTCATTCGC). The primers for *Cd8* are CD8-for (TGGCCTCACCGTTGACCCG) and CD8-Rev (CAGATCCTGCTGTTTCCACCT). All primer pairs were designed so that amplification occurs across an intron thereby allowing the distinction between amplification from genomic DNA and cDNA.

### Creation of the m1928z CAR

RNA was isolated from the 1D3 hybridoma (ATCC, Manassas, VA) that secretes a rat anti-mouse CD19 antibody. Degenerate oligos were used to amplify the immunoglobulin heavy (IgH) and light chain (IgL) rearrangements [18], [19]. Overlap PCR was used to create a single-chain fragment variable (scFv) composed of (5' to 3') a mouse CD8 signal peptide, IgH rearrangement, glycine-serine linker, and IgL rearrangement [20]. In addition, the mouse CD8 transmembrane region, mouse CD28 signal transduction domain, and mouse CD3ζ cytoplasmic domains were cloned from C57BL/6 mouse splenocyte mRNA. A series of overlap PCR steps were performed to fuse the scFv to the CD8, CD28, and CD3ζ domains. Lastly, to assess gene-transfer efficiency and monitor the adoptively transferred T cells we performed an overlap PCR to create a bicistronic genetic construct (GL-2A-m1928z) that coexpresses GFP and the m1928z CAR by using a 2A peptide sequence. This genetic construct was cloned into the vector backbone SFG, which is a Moloney murine leukemia-based retroviral vector [21].

### Mouse T cell activation, transduction, and adoptive transfer

Spleens were harvested from sacrificed mice. T cells were enriched from splenocytes by passage over a nylon wool column (Polysciences, Warrington, PA) or with a negative T cell isolation kit (Invitrogen). Mouse T cells were then activated with CD3/CD28 Dynabeads (Invitrogen) and cultured in the presence of human IL2 at 30 IU/mL (R & D Systems, Minneapolis, MN). Enrichment and activation was performed according to the manufacturers' instructions. Spinoculations were done twice with retroviral supernatant prepared from Phoenix-E packaging cells. Gene-transfer is estimated by the percentage of GFP^+^ T cells detected by flow cytometry or by measuring Vector Copy Number with quantitative PCR as described [5]. Further details regarding the isolation, activation, and transduction of mouse T cells are available [22]. For adoptive transfer of CD19 CAR-targeted T cells mice were injected with intraperitoneal (IP) cyclophosphamide followed 1 day later by an intravenous (IV) injection of T cells.

### Cytokine Detection

Transduced T cells were incubated for one day with 3T3 cells transduced with mouse CD19 (3T3-mCD19). Culture medium was complete RPMI supplemented with IL2 at 30 IU/mL. Culture medium was sampled one day after co-culture with T cells and 3T3-mCD19. Sampled tissue culture media was incubated with a Milliplex multi-analyte panel for mouse cytokines (EMD Millipore, Billerica, Massachusetts) and analyzed on a Luminex 100 system.

### Flow Cytometry

The following antibodies, with their clones listed parenthetically, were obtained from Ebioscience (San Diego, CA) or BD Biosciences (San Diego, CA), and used for flow cytometry: anti-B220 (RA3-6B2), anti-BP1 (6C3), anti-HSA (30-F1), anti-CD3 (500A2), anti-CD4 (RM4-5), anti-CD8 (53-6.7), anti-CD62L (MEL-14), anti-CD19 (1D3), anti-IgM (II/41) and anti-CD44 (IM7). In addition, the anti-CD43 antibody (clone 1B11) was obtained from Biolegend (San Diego, CA). Antibody staining of cultured T cells or tissues obtained from sacrificed mice was performed at 4°C with mouse Fc-block (Ebioscience) in 1% FBS in PBS. Stained-cells were washed once with 1% FBS in PBS before being processed through a 5-laser BD LSRII (BD Biosciences). Flow cytometry of blood cells was performed with a lyse-no wash preparation. Briefly, 25 µL of retro-orbital blood was incubated with antibodies for 25 minutes at 4°C. Afterwards, FACs Lysing Solution was added (BD Biosciences) and the cells were evaluated on the 5-laser BD LSRII. Unless stated otherwise, Countbright beads were used to calculate cell counts, which were done according to the manufacturer's instructions (Invitrogen). All flow cytometry data files were analyzed with FlowJo software (Tree Star, Ashland, OR).

### Statistics

Comparison of means was performed using t tests or ANOVA testing, depending on the number of groups being compared. For comparison of survival among treatment groups we used log-rank tests. All statistical analyses were performed with Prism 5 software (Graphpad, La Jolla, CA).

## Results

### Development of an immunocompetent mouse model for B-ALL

**Figure 1:**
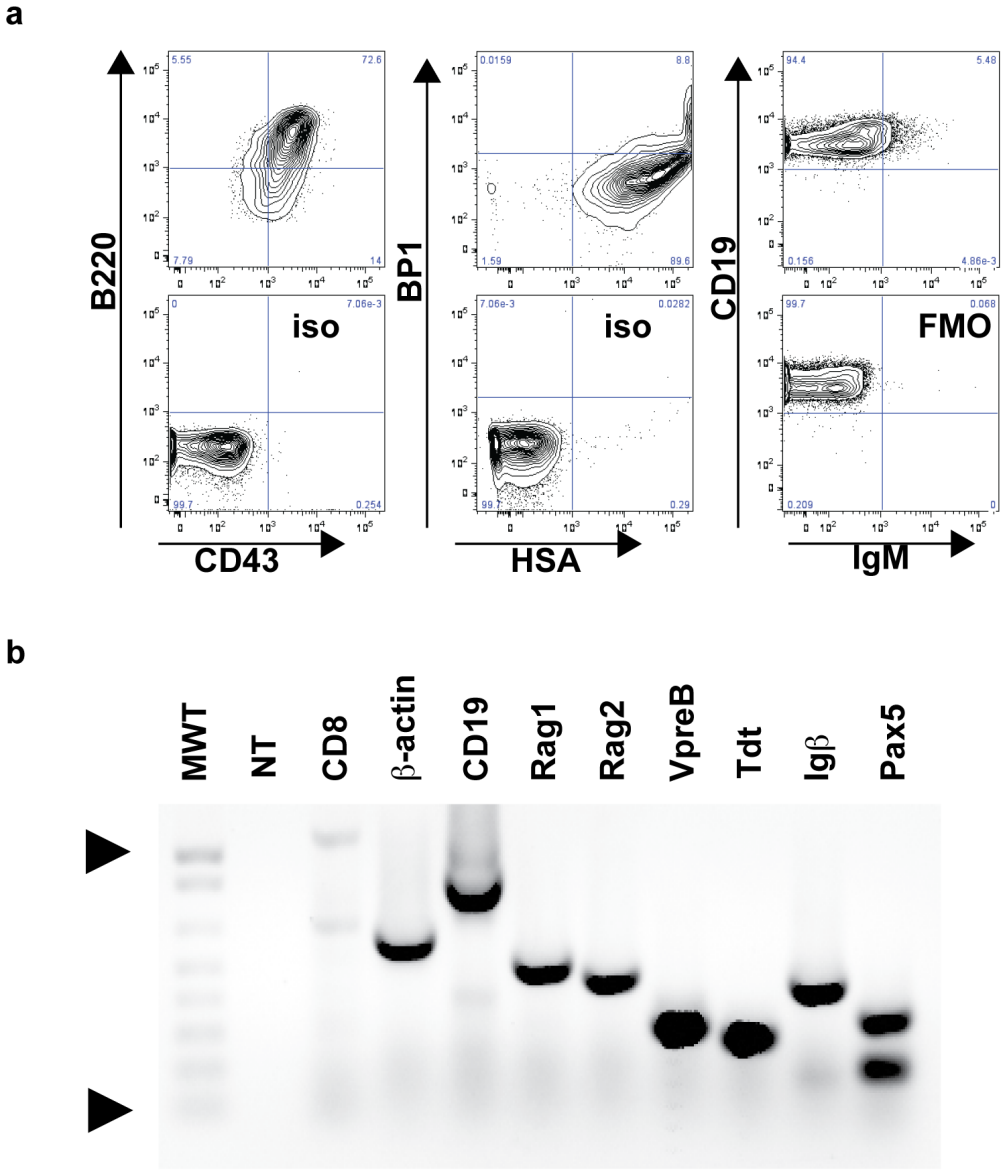
Immunophenotyping (a) and gene-expression (b) of Eμ-ALL01 cells. Immunophenotyping was performed with the antibodies listed on the axes. Cells were first gated within a live lymphoid gate on a scatter plot. The bottom panels are isotype (iso) or fluorescence minus one (FMO) controls. (b) RT-PCR was performed for the listed genes and electrophoresed through a 1% agarose gel and stained with Ethidium Bromide. No-template (NT) and *Cd8*, which is expressed usually in T cells but not B cells, are included as negative controls. MWT refers to the 1 Kb Plus DNA ladder (Invitrogen). The triangles point to the 1 Kb (top) and 100 bp fragments.

To develop a syngeneic and immunocompetent mouse model of B-ALL, we isolated malignant progenitor B cells from a lymph node of a female Eμ-myc C57BL/6 transgenic mouse with progressive disease (Supplementary Figure 1). The isolated cells were cultured until transformation and subsequently named Eμ-ALL01. Cell lines have been previously isolated from Eμ-myc transgenic mice, but like other spontaneous tumors isolated from transgenic mice, they are heterogeneous ranging from surface IgM (sIgM) negative progenitor B cells to sIgM^+^ mature B cells, and can appear as lymphomas or leukemias or plasmacytomas [15], [23]–[25]. Therefore, we characterized the immunophenotype [17] and gene-expression pattern to demonstrate that the cells have a progenitor B cell phenotype (B220+CD19+CD43+BP1+HSA-IgM-, Figure 1).

**Figure 2:**
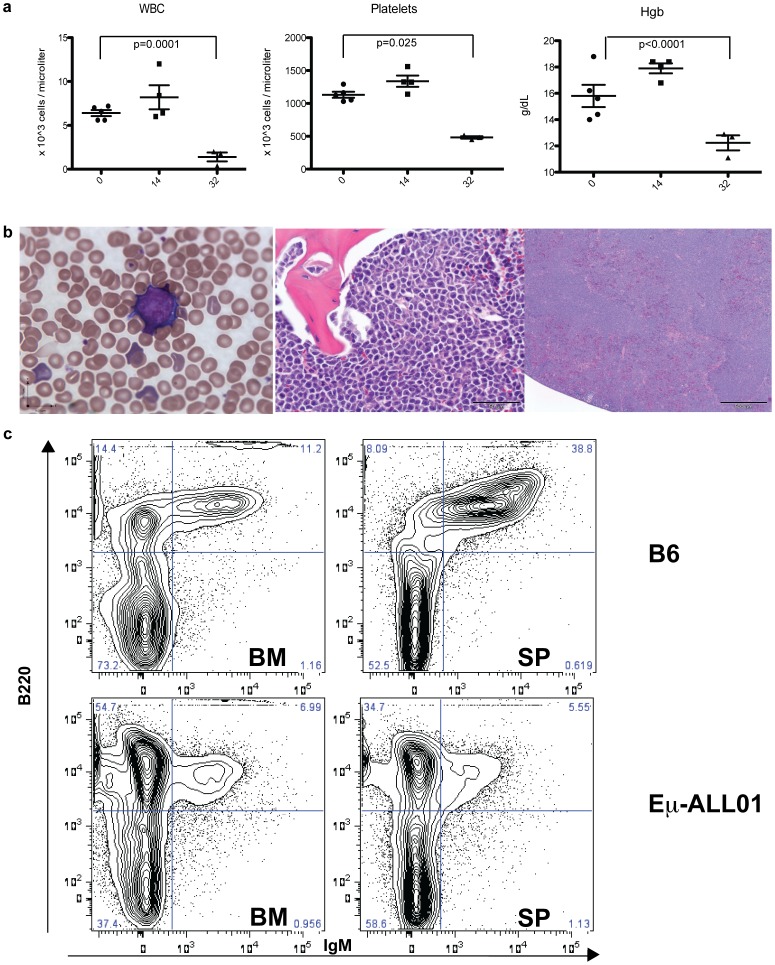
Progressive disease in mice after injection with Eμ-ALL01 tumor cells. (a) Retro-orbital blood was collected from 5 mice on the days indicated after Eμ-ALL01 injection and analyzed serially over time with an A^C^T diff cell counter (Beckman Coulter, Brea, CA). White cell count (WBC), Hemoglobin (Hgb), and Platelets were measured and statistically analyzed with t-tests, which were non-significant for Day 0 and Day 14 means. Comparison of the Day 0 and Day 32 means by t-tests were all significant (p values are noted on graph). Error bars are the standard error of the mean (SEM). The mice were sacrificed 4 weeks after injection with Eμ-ALL01 because of clinical deterioration and retro-orbital blood, bone marrow, and spleen were harvested for anatomical (b) and cellular (c) analyses, with representative images displayed. (b) Blood and tissue were used to prepare peripheral smears (left-panel) and H&E stained slides of sections of the bone marrow (middle-panel) and spleen (right-panel). (c) Single-cell suspensions were prepared from bone marrow (BM) and spleen (SP) and then incubated with antibodies specific for B220 and IgM. The panels on the top are cells isolated from disease-free, wild-type C57BL/6 mice (B6) and the panels on the bottom were injected with the Eμ-ALL01 cells. Cells displayed have been gated on live lymphoid cells.

We injected the transformed cells intravenously (IV) into wild-type C57BL/6 (B6) mice to determine if the subsequent clinical phenotype resembled B-ALL. Around two to four weeks after IV injection, all mice were pancytopenic and died of bone marrow (BM) failure secondary to progressive BM infiltration by mononuclear, homogenous B lymphoid cells that have the same immunophenotype (B220^+^CD43^+^IgM^−^) as Eμ-ALL01 cells (Figure 2 and Supplemental Figure S1). While mice die around two to four weeks after injection with Eμ-ALL01, during the first two weeks there were no overt signs of disease or distress, and based on complete blood counts there was no evidence of hematologic complications (Figure 2). Furthermore, necropsies showed variable signs of Eμ-ALL01 infiltration into the spleen and lymph node, which suggests that the tumor cells homed to the BM to proliferate until BM function was compromised, while their presence in other lymphoid organs represents “spill-over” from filled marrow space. The immunophenotype, gene-expression, and clinical phenotype are supportive of Eμ-ALL01 as a syngeneic, immunocompetent mouse model for B-ALL.

### Syngeneic T cells expressing a CD19-targeted chimeric antigen receptor mediate in vitro cytolysis of malignant B cell targets

**Figure 3:**
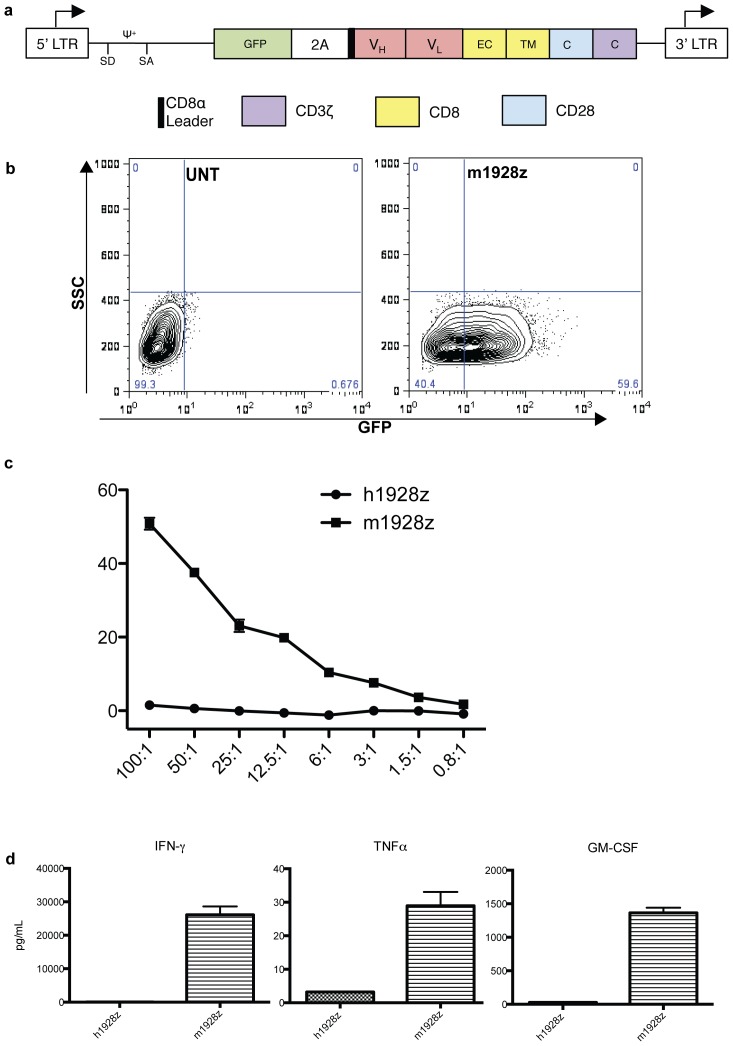
Generation and *in vitro* function of the m1928z CAR. (a) Schematic of the genetic construct GL-2A-m1928z for the reporter gene (GFP) and CAR (m1928z). Depicted are the packaging signal ψ, splice donor (SD), splice acceptor (SA), the V_H_ and V_L_ regions of the scFV, and the extracellular (EC), transmembrane (TM), and cytosolic (C) regions. (b) GFP-expression in mouse T cells after transduction with GL-2A-m1928z retroviral supernatant (right-panel). The left-panel displays untransduced (UNT) T cells as a control. The gene-transfer efficiency, estimated as the GFP+ population, is derived from a single experiment with a double-transduction of a bulk population of mouse T splenocytes as described [22]. (c) Cytotoxic T lymphocyte antigen-specific killing was evaluated with a Chromium release assay [39]. Target cells (EL4-mCD19) are EL4 cells retrovirally transduced with mouse CD19. Effector cells are T cells transduced with GL-2A-m1928z. Control effector cells were transduced with GL-2A-h1928z since it is identical to GL-2A-m1928z except for the scFv, which is derived from an anti-human CD19 antibody [5]. Effector to target ratio (x-axis) are based on the number of GFP^+^ T cells to EL4-mCD19 cells and were performed in triplicate. Killing efficiency (y-axis) was calculated as described [39]. (d) Cytokine secretion by m1928z and h1928z T cells was evaluated after stimulation with 3T3-mCD19 cells. Stimulation with 3T3-mCD19 cells was performed in triplicate and supernatants were obtained 1 day after stimulation. Each supernatant was evaluated for cytokines also in triplicate (no dilution, 3×dilution, and 9×dilution). Error bars represent the SEM.

We created the m1928z chimeric receptor by cloning the *IgH* and *IgL* rearrangements from a hybridoma that secretes a rat anti-mouse CD19 IgG2a antibody (Figure 3a). The GL-2A-m1928z bicistronic construct includes a GFP reporter protein and the m1928z CAR, which includes the anti-CD19 scFV, Glycine-Serine Linker, CD8 transmembrane region, CD28, and CD3ζ signaling elements. Retroviral transduction of mouse T cells with this construct results in efficient gene-transfer (Figure 3b). Furthermore, in a cytotoxic T lymphocyte (CTL) assay the CD19 CAR-targeted T cells, compared to control T cells, efficiently lyse EL4 targets transduced with mouse CD19 (Figure 3c). T cells gene-targeted with m1928z also produce abundant effector cytokines compared to control T cells (Figure 3d).

### CD19 CAR-targeted T cells eradicate Eμ-ALL01 in vivo

**Figure 4:**
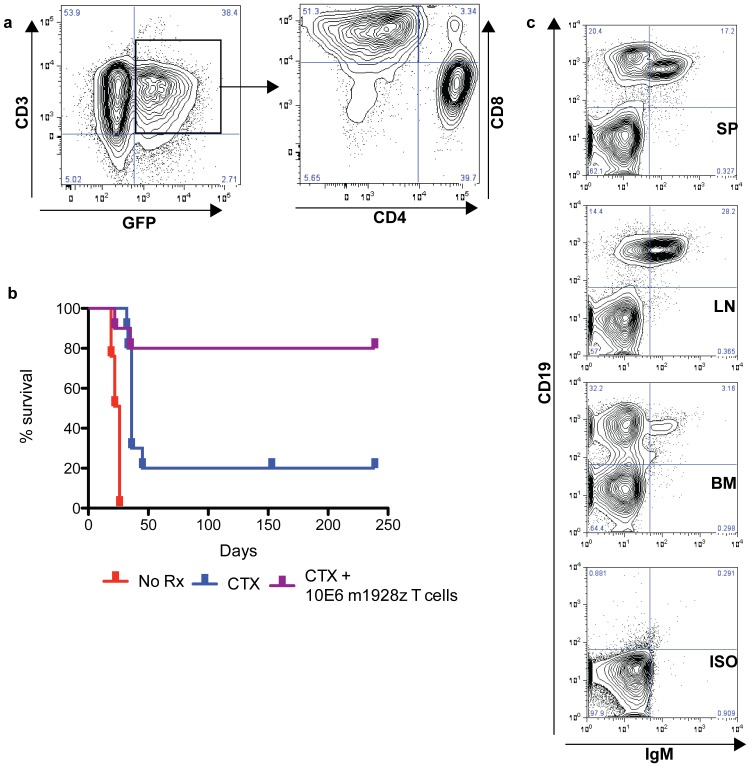
Adoptive transfer of CD19 CAR-targeted T cells into mice with leukemia. (a) Flow cytometry of bulk mouse T splenocytes double-transduced with GL-2A-m1928z. (b) Survival curve of mice injected with Eμ-ALL01 and not treated (No Rx), treated only with cyclophosphamide (CTX), or treated with cyclophosphamide (100-200 mg/kg IP) and CD19 CAR-targeted T cells (CTX+m1928z), displayed in (a). This data (n = 28) is pooled from 2 independent experiments. Another study (Supplemental Figure S2) confirms that cyclophosphamide and control T cells mediate no survival advantage. (c) Flow cytometry of cells from the BM, spleen (SP), and lymph node (LN) of a single mouse treated with cyclophosphamide but sacrificed due to clinical deterioration. Splenocytes were also stained with isotype antibodies (ISO) as a control.

We used our immunocompetent mouse model of B-ALL to evaluate critical factors involved in the killing of targets *in vivo*, such as homing, conditioning chemotherapy, immunophenotype, T cell dose, and persistence. Eμ-ALL01 targets were injected into B6 mice for one week, during which we transduced T cells with the m1928z CAR and expanded them *ex vivo*. Before treatment, we evaluated the T cells by flow cytometry (Figure 4a). We found the T cells to be a heterogeneous population of CD4 and CD8 T cells with a transduction efficiency of 38.4% for the m1928z CAR [26].

One week after injection of the Eμ-ALL01 tumor cells, mice were given a single intra-peritoneal injection of cyclophosphamide (Figure 4b). One-half of the mice injected with cyclophosphamide were followed without further treatment to evaluate for chemotherapy-related killing of tumor cells. The other half were intravenously injected with 10×10^6^ GFP+ CD19 CAR-targeted T cells. As in adults with B-ALL, there does appear to be a small benefit to chemotherapy. In the mice treated with chemotherapy alone, about 20% have long-term survival and the remaining mice have a very short delay until death. Flow cytometry demonstrates that these mice die with abundant tumor cells in their bone marrow and spleen (Figure 4c). This suggests that these mice die of progressive disease and not from complications related to the chemotherapy.

Mice treated with both cyclophosphamide and CD19 CAR-targeted T cells have significantly enhanced survival compared to mice treated with cyclophosphamide alone (p = 0.026, Figure 4b). The only deaths in the mice treated with cyclophosphamide and CAR-modified T cells came early (<40 days after tumor injection) and appeared at a similar time-point as in control mice treated with chemotherapy alone. Remaining mice have been monitored for almost one year after treatment with no evidence of complications from the gene-modified T cell infusions. No mice have been sacrificed in this group for any reason other than recurrent disease or interim analyses.

### CD19 CAR-targeted T cells eradicate normal B cells in vivo

**Figure 5:**
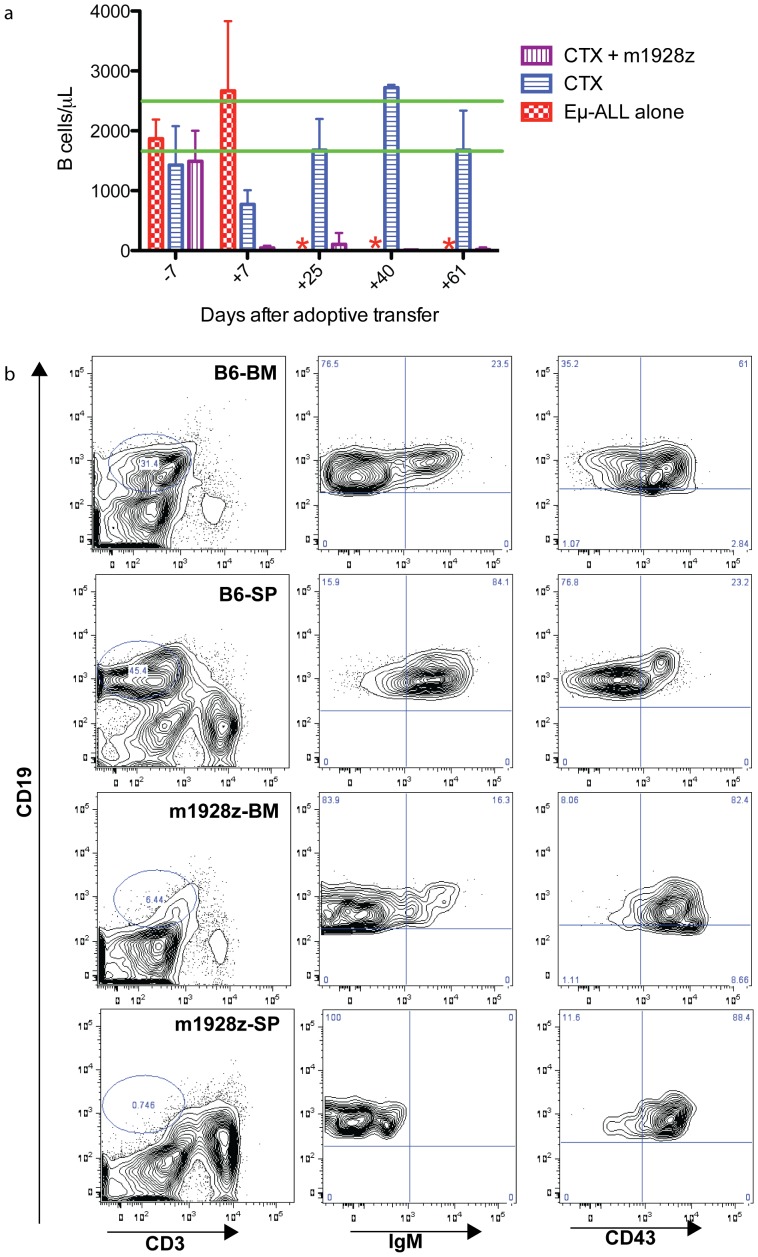
Persistent *in vivo* B cell targeting and regeneration after CD19 CAR-targeted T cell transfer. (a) Peripheral blood B cell counts from mice (n = 25) treated with 100 mg/kg IP cyclophosphamide (CTX) and/or CD19 CAR-targeted T cells was measured on the days listed on the x-axis. All groups were infused with Eμ-ALL01 tumor cells and the CTX group is treated with chemotherapy alone, CTX+m1928z group is treated with both chemotherapy and T cells, while the Eμ-ALL01 group is untreated. The red asterisks note time points when no samples were available since all the mice in the Eμ-ALL01 group had died. The green lines mark the range of the 95% confidence interval for the peripheral B cell count in wild-type untreated B6 mice. Error bars are the SEM. Statistical analyses were performed on both treatment groups using t tests for Days 7, 25, 40, and 61. For every one of these comparisons p<0.01. Retro-orbital blood was collected on the indicated days (Day 0 being adoptive transfer of T cells) and analyzed with an A^C^T diff cell counter and then incubated with anti-B220, anti-CD19, and anti-CD3 antibodies. The number of B cells was calculated as the concentration of cells multiplied by the frequency of CD19^+^ cells. (b) BM and spleen cells were isolated from a C57BL/6 mouse (B6) as a control, or a single mouse that had been injected with Eμ-ALL01 and treated eight-months prior with cyclophosphamide (100 mg/kg IP) and CD19 CAR-targeted T cells (m1928z). Cells were stained with anti-CD19, anti-CD3, anti-IgM, and anti-CD43 antibodies. In every row, the two far-right panels are derived from the CD19+ gate of the left-panel.

We assayed for in vivo CAR-modified T cell function by measuring the number of B cells in the peripheral blood of treated and control mice. As early as one week after treatment with cyclophosphamide and CD19 CAR-targeted T cells there is a loss of peripheral B cells, which is sustained for at least two months (Figure 5a). In contrast, there is a moderately decreased B cell count in the cyclophosphamide only group, which normalizes by 4 weeks after adoptive transfer. A representative flow cytometry plot of peripheral blood from mice that received cyclophosphamide and CD19 CAR-targeted T cells is shown to have T cells, but not B cells (Supplemental Figure S3a). Furthermore, in a chromium release assay we detected persistent CD19 CAR-targeted T cell function by culturing splenocytes, from mice adoptively transferred with these T cells one month prior, with EL4-mCD19 targets (Supplemental Figure S3b).

The short-lived decrease in B cells and poor survival of mice treated with cyclophosphamide alone suggests that infused CD19 CAR-targeted T cells kill residual malignant and normal B cells. Furthermore, the loss of B cells for two months suggest that the adoptively transferred T cells are functional long-term. However, claims of aplasia are incomplete without evaluating the bone marrow and spleen, so we sacrificed a mouse with a loss of B cells in the blood at eight months post treatment. The spleen and BM of a wild-type mouse has normal compartments of progenitor and mature B cells (Figure 5b). However, there is a dramatic reduction of B cells in the BM and spleen of a mouse infused with CD19 CAR-targeted T cells. The B cells that remain are IgM- and CD43+, which is an immunophenotype consistent with progenitor B cells (Figure 5b).

### Conditioning chemotherapy and T cell dose dependence of CAR-modified T cell persistence and B cell targeting

**Figure 6:**
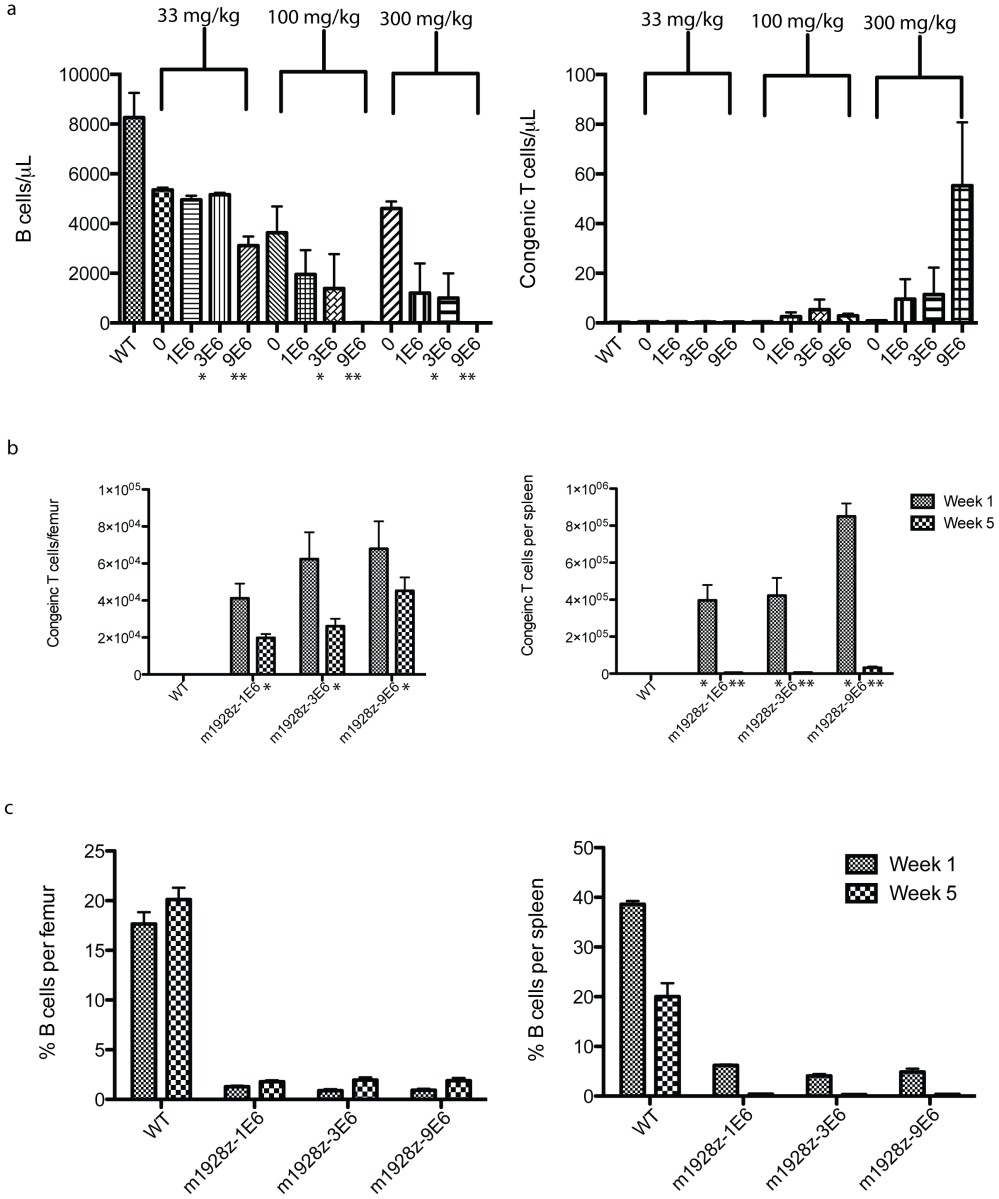
CD19 CAR-targeted T cell dependence on conditioning chemotherapy and T cell dose. T cells were transduced with m1928z and infused at increasing doses (noted on axes) into congenic mice (n = 40) that had been conditioned with increasing doses of cyclophosphamide (noted above columns). Peripheral blood B and congenic T cells were evaluated by flow cytometry for B and T cell markers 4 weeks after adoptive transfer. (b and c) A repeat experiment (n = 44) was done while holding cyclophosphamide conditioning constant (300 mg/kg), but with increasing T cell doses. Mice were sacrificed 1 and 5 weeks after adoptive transfer with CD19 CAR-targeted congenic T cells and flow cytometry for B cell (B220) and congenic T cells (Thy1.1/Thy1.2) was performed on single-cell suspensions of the BM and spleen. All counts and percentages were done with Countbright beads (Invitrogen). Statistical significance (p <0.05) was calculated by one-way ANOVA and is denoted by asterisks. For all sections of this figure error bars represent the SEM.

Studies have demonstrated that some form of conditioning therapy is required before gene-modified T cell infusion [27]–[29]. We performed a dose escalation of T cells and/or conditioning chemotherapy to determine if maximizing the in vivo E∶T ratio would enhance B cell eradication and T cell persistence (Figure 6). Due to concerns for possible immune rejection of GFP+ T cells we modified the genetic constructs by deleting the GFP reporter gene. To monitor transduction efficiency we performed qPCR and/or Protein-L staining by flow cytometry [30]. To monitor transduced T cells we used Thy1.1/Thy1.2 congenic markers to differentiate between host T cells and donor CD19 CAR-transduced T cells.

Wild-type mice were injected with increasing doses of cyclophosphamide (0 to 300 mg/kg) and increasing doses (1 to 9×10^6^ T cells) of congenic CD19-targeted T cells (Figure 6a). T cell persistence and B cell targeting was evaluated in the blood at 1-month post-T cell infusion. As the dose of cyclophosphamide is increased, the number of circulating B cells decreased 600-fold and the number of adoptively transferred T cells increased 6-fold. Furthermore, one-way ANOVA of the samples revealed that increasing cyclophosphamide, while holding the CAR-modified T cell dose constant at 3×10^6^ or 9×10^6^, increased B cell eradication at statistically significant levels, p = 0.04 and p<0.0001, respectively.

CAR-modified T cells appeared to be enhanced by increasing the T cell dose but the differences were not statistically significant (Figure 6a). However, since the number of CAR-modified T cells in the blood may not reflect their accumulation in the BM or spleen we sacrificed mice in a separate experiment at 1 and 5 weeks post-T cell infusion (Figure 6). Conditioning was held constant with 300 mg/kg IP cyclophosphamide since this dose had optimal B cell eradication by CAR-modified T cells. We detected high levels of persisting congenic T cells in the BM (48–67% from 1 to 5 weeks), but not in the spleen (1.5–3.7%). Furthermore, five weeks after infusion with the lowest T cell dose (1×10^6^) the entire BM compartment contained 3.1×10^5^ congenic T cells (31% of the original T cell dose), which was modestly lower than the 7.1×10^5^ congenic T cells (8% of the original T cell dose) in the BM after infusion with 9×10^6^ CAR-modified T cells (Table 1). Thus, despite a 9-fold difference in CD19 CAR-targeted T cell dose, the number of T cells persisting 5 weeks after adoptive transfer in the BM was similar in both groups.

**Table 1:** Antigen-dependence of CD19 CAR-targeted T cells in the BM.

			Week 1			
	B lineage cells/femur				Donor Tcells/femur	
**1×10^6^**	**3.0×10^6^**	**9.0×10^6^**		**1×10^6^**	**3×10^6^**	**9×10^6^**
2.0×10^5^	3.1×10^4^	6.2×10^4^		5.8×10^4^	1.5×10^4^	3.2×10^4^
1.5×10^5^	1.6×10^5^	1.6×10^5^		1.6×10^4^	7.5×10^4^	6.5×10^4^
1.1×10^5^	1.1×10^5^	1.4×10^5^		3.1×10^4^	6.1×10^4^	1.1×10^5^
1.0×10^5^	1.1×10^5^	9.8×10^4^		4.4×10^4^	5.7×10^4^	9.8×10^4^
1.1×10^5^	7.7×10^4^	6.7×10^4^		5.6×10^4^	1.0×10^5^	4.0×10^4^
			**E∶T ratio**	**1∶4.0**	**1∶1.7**	**1∶1.7**
			**% Dose**	**65.1**	**32.9**	**11.9**

			Week 5			
	B lineage cells/femur				Donor Tcells/femur	
**1 × 10^6^**	**3.0×10^6^**	**9.0×10^6^**		**1×10^6^**	**3×10^6^**	**9×10^6^**
2.3×10^5^	2.6×10^5^	2.7×10^5^		1.7×10^4^	1.9×10^4^	2.6×10^4^
2.5×10^5^	2.2×10^5^	1.8×10^5^		1.5×10^4^	2.9×10^4^	4.1×10^4^
2.4×10^5^	1.9×10^5^	2.3×10^5^		1.9×10^4^	3.3×10^4^	5.5×10^4^
2.3×10^5^	1.8×10^5^	1.7×10^5^		2.7×10^4^	1.5×10^4^	5.8×10^4^
1.6×10^5^	2.5×10^5^			2.0×10^4^	3.5×10^4^	
			**E∶T ratio**	**1∶11.7**	**1∶9.1**	**1∶5.5**
			**% Dose**	**31.2**	**13.8**	**7.9**

B and congenic T cell counts from the BM of mice treated with cyclophosphamide (300 mg/kg) and increasing doses (labeled in bold) of congenic m1928z T cells. Mice were sacrificed 1 and 5 weeks after adoptive transfer with congenic T cells and flow cytometry for B cell (B220) and congenic T cells (Thy1.1/Thy1.2) was performed on single-cell suspensions of the BM. Counts were facilitated with Countbright beads (Invitrogen). E∶T ratio is the ratio of congenic donor T cells to B cells in the BM.% Dose is the m1928z T cell dose divided by the total number of BM congenic T cells, estimated by multiplying the femoral congenic T cell count by 15.8 [5].

B cells were dramatically reduced in the BM (2.2×10^5^ B cells/femur) and virtually absent from the spleen (1.2×10^4^ B cells/spleen). We have demonstrated that the CD19+ cells that remain are progenitor B cells and that this reduction can be long lasting (at least 8 months) (Figure 5). Persistence of CAR-modified T cells and progenitor B cells in the BM suggests an attempt to repopulate the B cell compartment is thwarted by CD19 CAR-targeted T cells that eradicate progenitor B cells before they complete development. B and congenic T cell counts allow calculation of congenic T cell to B cell ratios in the BM (Table 1). These results suggest that the minimum E∶T ratio needed to maintain the aplasia and prevent B cell repopulation in the periphery is 1 donor T cell for every 12 B lineage cells (Table 1).

### Long-term functional persistence of CD19-targeted T cells requires only the adoptive transfer of CD8+ gene-targeted T cells and is independent of TCR signaling

**Figure 7:**
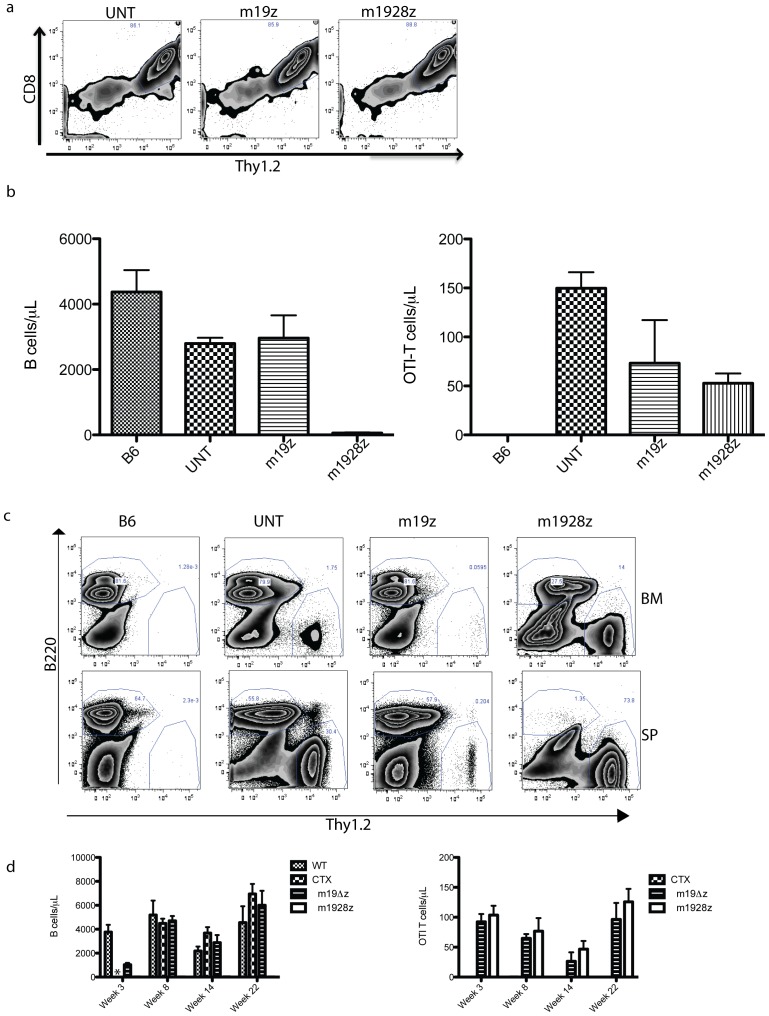
Adoptive transfer of CD8+ CD19 CAR-targeted T cells alone is sufficient for long-term persistence and B cell eradication. (a) OTI Thy1.2+ T cells were transduced with the m1928z CAR or the m19z CAR, which has a CD3ζ signal transduction domain but lacks the CD28 signal transduction domain. Untransduced T cells (UNT) are mock-transduced cells and are included as a negative control. Donor T cells (5×10^6^) were then adoptively transferred into congenic Thy1.1 mice (n = 19) 1 day after treatment with cyclophosphamide (300 mg/kg). (b) Peripheral blood B and OTI T cell counts were evaluated by flow cytometry 4 weeks after adoptive transfer. One-way ANOVA was done for the treatment groups' (UNT, m19z, m1928z) B cell counts (p = 0.0001) and also OTI T cell counts (p = 0.03). Error bars are the SEM. (c) A few of the mice in each group were sacrificed 7 weeks after adoptive T cell transfer. BM and spleen cells were analyzed by flow cytometry for B and congenic T cell markers. A representative plot is included from each group. Displayed cells were gated on Live and DUMP-negative cells, which were characterized with Mac1, Gr1, NK1.1, and Ter119 antibodies. (d) In another study (n = 17), peripheral blood counts of B and congenic T cells were performed from 3–22 weeks after adoptive transfer. This study included groups of control mice that were untreated wild-type (WT), or treated with cyclophosphamide alone (CTX), or treated with T cells modified with the m19Δz CAR, which lacks any signaling element. Statistical analyses (t tests) of the treatment groups (m19Δz and m1928z) were significant (p<0.05) when done on the B cell counts but not the OTI T cell counts (p = 0.47). Error bars are the SEM. * is Not Done.

We evaluated the immunophenotype of congenic CAR-modified T cells in the BM from the experiment described in Figure 6. We identified the appearance of central-memory T cells (CD44+ CD62L+) [31] and nearly all of these CD62L+ congenic cells are within the CD8+ T cell subset (Supplementary Figure 4). The appearance of CD8+ central memory T cells suggests that the infusion of CD8+ T cells alone may be sufficient for long-term B cell eradication. To evaluate this possibility we infused only CD8+ gene-modified T cells. However, to exclude any role that TCR activation may have on CAR-modified T cells we used T cells from OTI/Rag2−/− mice as the donor of CAR-modified T cells [32]. These mice have a *Tcr* transgene specific for ovalbumin and it is CD8-restricted, while the *Rag2* genetic deletion renders their T cells incapable of rearranging other *Tcr* gene segments. In addition to the second-generation m1928z CAR, groups included OTI T cells genetically targeted with a first-generation CAR (m19z) or mock transduced (UNT). Peripheral blood was evaluated for B cells and OTI T cells 4 weeks after cyclophosphamide and CD19 CAR-targeted T cell infusion (Figure 7). A dramatic reduction of B cells is evident only in mice treated with m1928z T cells.

Six weeks after infusion of CD8+ CAR-modified OTI T cells we sacrificed some of the mice in each group to evaluate B cells and donor T cells in the BM and spleen. A loss of B cells in the spleen was evident only in the m1928z group (Figure 7). The BM of these mice had a dramatic reduction of B cells, while the remaining B cells (3.2 to 3.6×10^5^ B cells/femur) likely represented progenitor B cells attempting to re-populate the B cell compartment as in Figure 5. Follow-up blood analyses in a separate study demonstrated persistent B cell aplasia and OTI-T cells, compared to control groups, nearly six months after treatment with cyclophosphamide and CD19 CAR-targeted OTI T cells (Figure 7).

## Discussion

### A model for the treatment of B-ALL using CD19-targeted T cells

B-ALL in adults remains a challenge to medical oncologists because of poor overall survival. We currently have three open clinical trials for adults with B cell malignancies: two for CLL and the other for B-ALL. We are using a second-generation CAR (19-28z) for our clinical trials based on results showing increased B cell tumor eradication compared to a first generation CAR [7].

We expect that our clinical trials will lead to new questions that need to be evaluated in pre-clinical models. The development of our immunocompetent mouse model of B-ALL was initiated because immunodeficient mouse models do not account for how T cell function is affected by regulatory T cells, abundant endogenous target antigen (in the case of B cell target antigens), and possible immune rejection of adoptively transferred T cells by host NK and/or T cells. Our immunocompetent mouse model of B-ALL offers several advantages compared to recent immunocompetent models that also use CD19 as a target [27], [28], [33]. First and foremost is that the Eμ-ALL01 tumor model resembles B-ALL by immunophenotype, gene-expression, and pathology (Figures 1 and 2). In addition, our model does not require a conditioning treatment before tumor infusion, suggesting that the tumor cells are not immunogenic [27]. Furthermore, the mice used in our studies are in-bred mice and not complex CD19 knockout/human CD19 transgenic double mutant mice, which can have B cell markers, counts, and humoral immunity perturbed compared to wild-type mice [28].

In our studies, m1928z T cells efficiently recognize and kill B cell targets, produce effector cytokines, and cure mice with B-ALL (Figure 4). These mice have dramatic reductions of B cells in the blood, spleen, and bone marrow (Figure 5). The few B cells that remain are in the BM and are CD43+, which suggests that there is an attempt to re-populate the B cell compartment but the B cells are targeted before they complete development (IgM+CD43−). Furthermore, these results suggest that there are two phases of CD19 CAR-targeted T cell function: 1) immediate tumor eradication, since mice die quickly if treated with chemotherapy alone (Figure 4), and 2) persistent targeting of re-emerging progenitor B cells, which may enhance long-term persistence and tumor surveillance. Furthermore, long-term persistence of CD19 CAR targeted T cells suggest that anti-CAR immune responses are not present, or at least not effective. However, this may not be true for other CARs, for example as seen with rejection of an anti-CD20 CAR [12], so anti-CAR immune responses should be included as part of future clinical trials to rule this out as a possible mechanism of limited CAR T cell persistence.

The evidence of long-term CD19 CAR targeted T cell persistence and B cell reductions suggest that clinical application of this technology could create a population of patients that are cured of their B lymphoid malignancy but have a long-term B cell aplasia. Indeed, there are anecdotal case reports of B cell aplasia in patients treated with CAR T cells targeted against CD19 [9], [10]. These patients were hypo-gammaglobulinemic, which would presumably place them at an increased risk for infection. As this therapy is adapted to more patients it represents a significant, potential limitation to its clinical success. It is possible that a dramatic survival advantage could be compromised by deaths from infections. Intermittent administration of antibiotics and/or intravenous immunoglobulins can be given, but they represent only temporary measures and may be ineffective over long durations. Therefore, incorporation of suicide genes into CD19 CAR targeted constructs represents one mechanism to address these long-term complications if they develop in patients [34], [35]. Activation of the suicide gene would lead to CAR T cell death and allow normal B cell recovery and recovery of protective immunoglobulins.

We evaluated how conditioning chemotherapy and T cell dose impacted T cell persistence and B cell eradication. Previous studies have shown that some form of conditioning treatment is required for optimal targeting of B cells [27]–[29]. Our results extend these observations by demonstrating that increasing conditioning chemotherapy increases B cell depletion, but increasing the dose of CAR-modified T cells does not, at least when preceded by optimal conditioning (Figure 6). Furthermore, there appears to be strong retention of CAR-modified T cells in the BM, but not spleen (Figure 6). It has been estimated [36] that approximately 1.5×10^5^ B cells are generated in the femur of a mouse every hour, which is similar to the total number of femoral B lineage cells we detected (0.97 to 2.2×10^5^) in mice treated with CD19 CAR-targeted T cells (Table 1). Therefore, the number of B lineage cells in the BM of treated mice likely reflects a balance of the development of new CD19+ progenitors B cells and their eradication by CD19 CAR-targeted T cells (Table 1). In addition, comparison of endogenous BM B cells and donor T cell numbers allowed us to estimate a minimum in vivo E∶T ratio (1 CD19 CAR-targeted T cell:12 B cells) required for persistent CAR-modified T cell function and retention (Table 1). This series of studies may allow us to finally estimate an optimal CAR-modified T cell dose for patients based on their tumor burden.

We evaluated the immunophenotype of adoptively transferred CD19 CAR-targeted T cells to determine if they are solely long-lived effector cells or if they may develop from central memory T cells (Supplemental Figure S4). From 1 to 5 weeks after transfer, there is a population of central-memory (CD44+CD62L+) T cells that appear in the BM. This suggests that a central memory population of CAR-modified T cells may serve as a reservoir for the development of new effector CD19 CAR-targeted T cells. Furthermore, most of the CD62L+ cells were restricted to the CD8+ T cell subset. We demonstrated that infusion of CD19 CAR-targeted CD8+ T cells alone was sufficient for long term B cell eradication and T cell persistence (Figure 7). This is in contrast to a previous study that suggested CAR+ T cell function required both CD4+ and CD8+ adoptive T cell transfer [37]. However, this study relied on immunodeficient mice and a limited, non-persistent antigen in mice (human erbB2). In this setting, CD4+ T cells may be necessary for optimal CAR+ T cell persistence and function. Since our model uses persistent, regenerating antigen, expressed by antigen presenting B cells in immunocompetent animals, the donor CAR-modified OTI T cells may not require CD19 CAR-targeted CD4+ T cell help. So these results may be applicable only in this setting and may or may not be translatable to other settings depending on the disease, target, and/or CAR construct. Regardless, these results argue that further studies are warranted involving CD4 and CD8 T cell subset contributions to CD19-targeted killing by CAR-modified T cells.

We did not evaluate for regulatory T cells since we detected long-term persistence and function of the adoptively transferred CD19 CAR targeted T cells. However, it is possible that regulatory T cells may be critical for regulating CAR T cell expansion and function so future studies will be directed at elucidating any potential role. Indeed, production of GMCSF (Fig. 3c) suggests that regulatory T cells could be activated by CAR T cells. However, if clinical application of CD19 CAR targeted T cells are compromised by regulatory T cells, as predicted by other pre-clinical studies [38], our observation of robust B cell depletion and CAR T cell persistence after infusion with CD8 T cells alone suggests a potential solution.

We believe that optimization of CD19 CAR-targeted T cell therapy for B-ALL requires an immunocompetent and physiologically relevant mouse model of B-ALL to address critical questions regarding CAR modified T cell therapy. We believe that these initial, and future, studies of mouse CD19 CAR-targeted T cells will ultimately benefit patients by allowing more extensive and rationale analyses than are possible with immunodeficient mouse models or patients.

## Supporting Information

Figure S1 **A progenitor B lymphoid tumor is isolated from the lymph node of an Eμ-myc transgenic mouse.** (a) A mouse moribund from disease was imaged with a 4.7-T 33-cm bore magnet imaging/spectroscopy system operating at 200 MHz while anesthetized with 2% isoflurane. The mouse was sacrificed and the axillary lymph node marked by the arrow was harvested. (b) A single cell suspension of this lymph node was analyzed by flow cytometry and used to culture the Eμ-ALL01 cell line.
(EPS)

Figure S2 **Survival curves for two groups of mice (n = 12) treated with cyclophosphamide and CD19-targeted gene-modified T cells.** Mouse T cells were retrovirally transduced with a construct encoding an m1928z CAR or m19Δz CAR, which is identical to m1928z except for lack of the CD28 and CD3ζ signal transduction domains. Mice were injected with 1×10^6^ Eμ-ALL01 tumor cells followed 1 week later by 300 mg/kg IP cyclophosphamide and then 1 day later by 3×10^6^ CAR-modified T cells. Log-rank Test for differences in survival were statistically significant (p = 0.0004).
(EPS)

Figure S3 **Peripheral B cell aplasias are mediated by CAR-modified anti-CD19 T cells.** (a) B and T cell populations in the retro-orbital blood of mice injected with Eμ-ALL01 tumor cells and then subsequently treated with cyclophosphamide (100 mg/kg IP) and/or m1928z-transduced T cells. Retro-orbital blood was isolated from mice two months after treatment and stained with anti-CD3, anti-CD19, and anti-IgM antibodies. The groups include C57BL/6 mice (B6) as controls, mice treated with cyclophosphamide alone (CTX), and mice treated with cyclophosphamide and m1928z T cells (CTX + m1928z). (b) T cells retain anti-CD19 targeted activity one month after adoptive transfer. Splenocytes were harvested from mice injected with cyclophosphamide (300 mg/kg IP) and either m19Δz, which lacks any signal transduction element, or m1928z T cells. The splenocytes were activated with CD3/CD28 beads (Invitrogen) and cultured for 5 days with cRPMI supplemented with IL2 (30 IU/mL). Splenocytes were then incubated, in triplicate, with radioactive-labeled EL4-mCD19 target cells at a 400∶1 ratio for 16 hours and% killing was calculated as described [5]. Error bars represent the SEM.
(EPS)

Figure S4 **Immunophenotype of post-transfer m1928z T cells.** (a) B6 (Thy1.2+) mice were conditioned with 300 mg/kg IP cyclophosphamide and 1 day later intravenously injected with 9×10^6^ m1928z-transduced Thy1.1+ T cells. Mice were sacrificed 1- and 5 weeks after adoptive transfer and femoral bone marrow was prepared and analyzed by flow cytometry. The CD44 and CD62L expression of Live, CD3+, Thy1.1+ T cells is depicted for one mouse, which is representative of the group of mice sacrificed at that time point. Pre are the m1928z-transduced Thy1.1+ T cells right before IV injection into mice. (b) CD8 and CD62L expression of Live, CD3+, Thy1.1+ T cells isolated from the BM of a mouse sacrificed 5 weeks after adoptive transfer with m1928z T cells.
(EPS)

## References

[1] Gokbuget N, Stanze D, Beck J, Diedrich H, Horst HA, et al (2012) Outcome of relapsed adult lymphoblastic leukemia depends on response to salvage chemotherapy, prognostic factors, and performance of stem cell transplantation. Blood 120: 2032–2041. 22493293 10.1182/blood-2011-12-399287

[2] Pui CH, Evans WE (2006) Treatment of acute lymphoblastic leukemia. N Engl J Med 354: 166–178. 16407512 10.1056/NEJMra052603

[3] Porter DL, Antin JH (1999) The graft-versus-leukemia effects of allogeneic cell therapy. Annu Rev Med 50: 369–386. 10073284 10.1146/annurev.med.50.1.369

[4] Passweg JR, Tiberghien P, Cahn JY, Vowels MR, Camitta BM, et al (1998) Graft-versus-leukemia effects in T lineage and B lineage acute lymphoblastic leukemia. Bone Marrow Transplant 21: 153–158. 9489632 10.1038/sj.bmt.1701064

[5] Brentjens RJ, Riviere I, Park JH, Davila ML, Wang X, et al (2011) Safety and persistence of adoptively transferred autologous CD19-targeted T cells in patients with relapsed or chemotherapy refractory B-cell leukemias. Blood 118: 4817–4828. 21849486 10.1182/blood-2011-04-348540 PMC3208293

[6] Brentjens RJ, Latouche JB, Santos E, Marti F, Gong MC, et al (2003) Eradication of systemic B-cell tumors by genetically targeted human T lymphocytes co-stimulated by CD80 and interleukin-15. Nat Med 9: 279–286. 12579196 10.1038/nm827

[7] Brentjens RJ, Santos E, Nikhamin Y, Yeh R, Matsushita M, et al (2007) Genetically targeted T cells eradicate systemic acute lymphoblastic leukemia xenografts. Clin Cancer Res 13: 5426–5435. 17855649 10.1158/1078-0432.CCR-07-0674

[8] Kochenderfer JN, Dudley ME, Feldman SA, Wilson WH, Spaner DE, et al.. (2011) B-cell depletion and remissions of malignancy along with cytokine-associated toxicity in a clinical trial of anti-CD19 chimeric-antigen-receptor-transduced T cells. Blood. 10.1182/blood-2011-10-384388 PMC3327450 22160384

[9] Kochenderfer JN, Wilson WH, Janik JE, Dudley ME, Stetler-Stevenson M, et al (2010) Eradication of B-lineage cells and regression of lymphoma in a patient treated with autologous T cells genetically engineered to recognize CD19. Blood 116: 4099–4102. 20668228 10.1182/blood-2010-04-281931 PMC2993617

[10] Kalos M, Levine BL, Porter DL, Katz S, Grupp SA, et al (2011) T cells with chimeric antigen receptors have potent antitumor effects and can establish memory in patients with advanced leukemia. Sci Transl Med 3: 95ra73. 10.1126/scitranslmed.3002842 PMC3393096 21832238

[11] Porter DL, Levine BL, Kalos M, Bagg A, June CH (2011) Chimeric antigen receptor-modified T cells in chronic lymphoid leukemia. N Engl J Med 365: 725–733. 21830940 10.1056/NEJMoa1103849 PMC3387277

[12] Jensen MC, Popplewell L, Cooper LJ, DiGiusto D, Kalos M, et al (2010) Antitransgene rejection responses contribute to attenuated persistence of adoptively transferred CD20/CD19-specific chimeric antigen receptor redirected T cells in humans. Biol Blood Marrow Transplant 16: 1245–1256. 20304086 10.1016/j.bbmt.2010.03.014 PMC3383803

[13] Savoldo B, Ramos CA, Liu E, Mims MP, Keating MJ, et al (2011) CD28 costimulation improves expansion and persistence of chimeric antigen receptor-modified T cells in lymphoma patients. J Clin Invest 121: 1822–1826. 21540550 10.1172/JCI46110 PMC3083795

[14] Davila ML, Brentjens R, Wang X, Riviere I, Sadelain M (2012) How do CARs work?: Early insights from recent clinical studies targeting CD19. OncoImmunology 1: 1577–1583. 23264903 10.4161/onci.22524 PMC3525612

[15] Corcoran LM, Tawfilis S, Barlow LJ (1999) Generation of B lymphoma cell lines from knockout mice by transformation in vivo with an Emu-myc transgene. J Immunol Methods 228: 131–138. 10556550 10.1016/s0022-1759(99)00094-0

[16] Li YS, Wasserman R, Hayakawa K, Hardy RR (1996) Identification of the earliest B lineage stage in mouse bone marrow. Immunity 5: 527–535. 8986713 10.1016/s1074-7613(00)80268-x

[17] Li YS, Hayakawa K, Hardy RR (1993) The regulated expression of B lineage associated genes during B cell differentiation in bone marrow and fetal liver. J Exp Med 178: 951–960. 8350062 10.1084/jem.178.3.951 PMC2191150

[18] Orlandi R, Gussow DH, Jones PT, Winter G (1989) Cloning immunoglobulin variable domains for expression by the polymerase chain reaction. Proc Natl Acad Sci U S A 86: 3833–3837. 2726754 10.1073/pnas.86.10.3833 PMC287235

[19] Wang YH, Stephan RP, Scheffold A, Kunkel D, Karasuyama H, et al (2002) Differential surrogate light chain expression governs B-cell differentiation. Blood 99: 2459–2467. 11895780 10.1182/blood.v99.7.2459

[20] Heckman KL, Pease LR (2007) Gene splicing and mutagenesis by PCR-driven overlap extension. Nat Protoc 2: 924–932. 17446874 10.1038/nprot.2007.132

[21] Riviere I, Brose K, Mulligan RC (1995) Effects of retroviral vector design on expression of human adenosine deaminase in murine bone marrow transplant recipients engrafted with genetically modified cells. Proc Natl Acad Sci U S A 92: 6733–6737. 7624312 10.1073/pnas.92.15.6733 PMC41403

[22] Lee J, Sadelain M, Brentjens R (2009) Retroviral transduction of murine primary T lymphocytes. Methods Mol Biol 506: 83–96. 19110621 10.1007/978-1-59745-409-4_7 PMC5003426

[23] Adams JM, Harris AW, Pinkert CA, Corcoran LM, Alexander WS, et al (1985) The c-myc oncogene driven by immunoglobulin enhancers induces lymphoid malignancy in transgenic mice. Nature 318: 533–538. 3906410 10.1038/318533a0

[24] Dierks C, Grbic J, Zirlik K, Beigi R, Englund NP, et al (2007) Essential role of stromally induced hedgehog signaling in B-cell malignancies. Nat Med 13: 944–951. 17632527 10.1038/nm1614

[25] Harris AW, Pinkert CA, Crawford M, Langdon WY, Brinster RL, et al (1988) The E mu-myc transgenic mouse. A model for high-incidence spontaneous lymphoma and leukemia of early B cells. J Exp Med 167: 353–371. 3258007 10.1084/jem.167.2.353 PMC2188841

[26] Sallusto F, Lenig D, Forster R, Lipp M, Lanzavecchia A (1999) Two subsets of memory T lymphocytes with distinct homing potentials and effector functions. Nature 401: 708–712. 10537110 10.1038/44385

[27] Kochenderfer JN, Yu Z, Frasheri D, Restifo NP, Rosenberg SA (2010) Adoptive transfer of syngeneic T cells transduced with a chimeric antigen receptor that recognizes murine CD19 can eradicate lymphoma and normal B cells. Blood 116: 3875–3886. 20631379 10.1182/blood-2010-01-265041 PMC2981541

[28] Pegram HJ, Lee JC, Hayman EG, Imperato GH, Tedder TF, et al (2012) Tumor-targeted T cells modified to secrete IL-12 eradicate systemic tumors without need for prior conditioning. Blood 119: 4133–4141. 22354001 10.1182/blood-2011-12-400044 PMC3359735

[29] James SE, Orgun NN, Tedder TF, Shlomchik MJ, Jensen MC, et al (2009) Antibody-mediated B-cell depletion before adoptive immunotherapy with T cells expressing CD20-specific chimeric T-cell receptors facilitates eradication of leukemia in immunocompetent mice. Blood 114: 5454–5463. 19880489 10.1182/blood-2009-08-232967 PMC2798862

[30] Zheng Z, Chinnasamy N, Morgan RA (2012) Protein L: a novel reagent for the detection of chimeric antigen receptor (CAR) expression by flow cytometry. J Transl Med 10: 29. 22330761 10.1186/1479-5876-10-29 PMC3299624

[31] Li N, Matte-Martone C, Zheng H, Cui W, Venkatesan S, et al (2011) Memory T cells from minor histocompatibility antigen-vaccinated and virus-immune donors improve GVL and immune reconstitution. Blood 118: 5965–5976. 21917752 10.1182/blood-2011-07-367011 PMC3228506

[32] Clarke SR, Barnden M, Kurts C, Carbone FR, Miller JF, et al (2000) Characterization of the ovalbumin-specific TCR transgenic line OT-I: MHC elements for positive and negative selection. Immunol Cell Biol 78: 110–117. 10762410 10.1046/j.1440-1711.2000.00889.x

[33] Cheadle EJ, Hawkins RE, Batha H, Rothwell DG, Ashton G, et al.. (2009) Eradication of Established B-cell Lymphoma by CD19-specific Murine T Cells is Dependent on Host Lymphopenic Environment and can be Mediated by CD4+ and CD8+ T Cells. J Immunother. 10.1097/CJI.0b013e318194a921 19242379

[34] Di Stasi A, Tey SK, Dotti G, Fujita Y, Kennedy-Nasser A, et al (2011) Inducible apoptosis as a safety switch for adoptive cell therapy. N Engl J Med 365: 1673–1683. 22047558 10.1056/NEJMoa1106152 PMC3236370

[35] Wang X, Chang WC, Wong CW, Colcher D, Sherman M, et al (2011) A transgene-encoded cell surface polypeptide for selection, in vivo tracking, and ablation of engineered cells. Blood 118: 1255–1263. 21653320 10.1182/blood-2011-02-337360 PMC3152493

[36] Opstelten D, Osmond DG (1983) Pre-B cells in mouse bone marrow: immunofluorescence stathmokinetic studies of the proliferation of cytoplasmic mu-chain-bearing cells in normal mice. Journal of immunology 131: 2635–2640. 6417229

[37] Moeller M, Haynes NM, Kershaw MH, Jackson JT, Teng MW, et al (2005) Adoptive transfer of gene-engineered CD4+ helper T cells induces potent primary and secondary tumor rejection. Blood 106: 2995–3003. 16030195 10.1182/blood-2004-12-4906

[38] Lee JC, Hayman E, Pegram HJ, Santos E, Heller G, et al (2011) In vivo inhibition of human CD19-targeted effector T cells by natural T regulatory cells in a xenotransplant murine model of B cell malignancy. Cancer Res 71: 2871–2881. 21487038 10.1158/0008-5472.CAN-10-0552 PMC3094720

[39] Gong MC, Latouche JB, Krause A, Heston WD, Bander NH, et al (1999) Cancer patient T cells genetically targeted to prostate-specific membrane antigen specifically lyse prostate cancer cells and release cytokines in response to prostate-specific membrane antigen. Neoplasia 1: 123–127. 10933046 10.1038/sj.neo.7900018 PMC1508130

